# Self-Assembled Superparamagnetic Iron Oxide Nanoclusters for Universal Cell Labeling and MRI

**DOI:** 10.1186/s11671-016-1479-5

**Published:** 2016-05-23

**Authors:** Shuzhen Chen, Jun Zhang, Shengwei Jiang, Gan Lin, Bing Luo, Huan Yao, Yuchun Lin, Chengyong He, Gang Liu, Zhongning Lin

**Affiliations:** Department of Microbiology and Immunology, Xiamen Medical College, Xiamen, 361008 China; State Key Laboratory of Molecular Vaccinology and Molecular Diagnostics, Center for Molecular Imaging and Translational Medicine, School of Public Health, Xiamen University, Xiamen, 361102 China; Sichuan Key Laboratory of Medical Imaging, Affiliated Hospital of North Sichuan Medical College, North Sichuan Medical College, Nanchong, 637007 China

**Keywords:** Nanoclusters, SPIO, MRI, Cell labeling, Biocompatibility

## Abstract

Superparamagnetic iron oxide (SPIO) nanoparticles have been widely used in a variety of biomedical applications, especially as contrast agents for magnetic resonance imaging (MRI) and cell labeling. In this study, SPIO nanoparticles were stabilized with amphiphilic low molecular weight polyethylenimine (PEI) in an aqueous phase to form monodispersed nanocomposites with a controlled clustering structure. The iron-based nanoclusters with a size of 115.3 ± 40.23 nm showed excellent performance on cellular uptake and cell labeling in different types of cells, moreover, which could be tracked by MRI with high sensitivity. The SPIO nanoclusters presented negligible cytotoxicity in various types of cells as detected using MTS, LDH, and flow cytometry assays. Significantly, we found that ferritin protein played an essential role in protecting stress from SPIO nanoclusters. Taken together, the self-assembly of SPIO nanoclusters with good magnetic properties provides a safe and efficient method for universal cell labeling with noninvasive MRI monitoring capability.

**Electronic supplementary material:**

The online version of this article (doi:10.1186/s11671-016-1479-5) contains supplementary material, which is available to authorized users.

## Background

Molecular imaging, such as magnetic resonance imaging (MRI), plays an important role in molecular or individual medicine, which enables us to visualize the molecular targets and diagnose complex diseases noninvasively [1, 2]. However, traditional MRI suffers from low sensitivity, and thus, the introduction of contrast agents is needed for histopathological examination and cell labeling and tracking [3]. Contrast agents have been proved to harbor the ability to improve the sensitivity of MRI [4]. Superparamagnetic iron oxide (SPIO) nanoparticles are typically MRI contrast agents and have also been widely used for cellular imaging [5–7], which are composed of either a magnetite (Fe_3_O_4_) or maghemite (γ-Fe_2_O_3_) core [8].

Generally, uncoated SPIO nanoparticles tend to aggregate when placed in an aqueous environment which limits their stability and the efficiency of cell labeling and tracking [9–12]. Thus, surface modification of SPIO nanoparticles is necessary for efficient cell labeling. To improve the efficiency of SPIO nanoparticles labeling cells, much effort on modification have been conducted, such as linking peptides or antibodies to the surface of SPIO nanoparticles [13–16]. Unfortunately, these approaches have some shortcomings, such as complexity of modifying procedures or low availability of cell labeling. Nowadays, a more promising approach is SPIO nanoparticles modification with polycations, such as poly-l-lysine (PLL) and polyethylenimine (PEI) [17, 18], both of which are considered to facilitate the cellular internalization as their positive charges. Additionally, low molecular weight PEI (2 kDa) presents lower cytotoxicity compared to high molecular weight PEI (25 kDa) [19, 20]. Herein, we hypothesized that amphiphilic low molecular weight PEI modified SPIO nanoclusters might to be a candidate for cellular MRI contrast agent as their positive charge and good biocompatibility.

In this study, we developed SPIO nanoclusters with a controlled clustering structure using alkyl-modified low molecular weight (2 kDa) PEI (Alkyl-PEI) to encapsulate SPIO nanoparticles for efficient cell labeling with MRI monitoring capability. The amine groups in the Alkyl-PEI are helpful for modification of various chemicals [21–23]. Furthermore, we evaluated the cell labeling efficiency of the nanoclusters using cellular MRI and Perl’s Prussian blue staining in three cell lines including mouse RAW264.7 macrophage cells, mouse NIH3T3 fibroblast cells, and human HepG2 hepatic cells. Notably, we systematically evaluated the cytotoxicity of the SPIO nanoclusters in these cells using many methods, including [3-(4,5-dimethylthiazol-2-yl)-5-(3-carboxymethoxyphenyl)-2-(4-sulfophenyl)-2H-tetrazolium] (MTS), lactase dehydrogenase (LDH) [24], flow cytometry (FCS), and western blotting assays. Our results showed that this low molecular weight Alkyl-PEI-modified SPIO nanocluster system has a great potential for universal cell labeling and tracking with excellent biocompatibility.

## Methods

### Synthesis and Characterization of SPIO Nanoclusters

Following a typical published protocol, we synthesized the SPIO core [6, 25]. Briefly, 20 ml benzyl ether were mixed with 2 mmol iron(III) acetylacetonate, 6 mmol oleic acid, 10 mmol 1,2-hexadecanediol, and 6 mmol oleylamine, which were then heat to 300 °C under protection with argon gas for 1 h. Next, the SPIO nanoclusters were prepared following a typical synthetic procedure with minor modification [6, 26, 27], and the SPIO and Alkyl-PEI (ratio 1:0.6) were dissolved in chloroform and ultrasonic treated for 24 h. Lastly, to obtain water-dispersible SPIO, chloroform was removed by rotary evaporation. The size and zeta potential of the nanoparticles were characterized using a Zetasizer Nano system (Nano-ZS, Malvern, UK). The overall morphology of the SPIO nanoclusters was imaged using transmission electron microscope (TEM) (Tecnai 20, FEI, USA). The hysteresis loop at 300 K was measured using a superconducting quantum interference device (SQUID) magnetometer (MPMS-XL-7, Quantum Design, USA).

### Cell Culture

RAW264.7 and NIH3T3 cells were cultured in DMEM (Gibco, USA), and HepG2 cells were grown in RPMI medium 1640 (Gibco) supplemented with 10 % fetal bovine serum, penicillin (100 U/ml), and streptomycin (100 U/ml) in humidified 5 % CO_2_ at 37 °C.

### Perl’s Prussian Blue Staining

Perl’s Prussian blue staining is applied for displaying ferric iron and ferritin protein. The three types of cells were seeded in 24-well plates at a density approximate 3 × 10^4^ cells/well. After labeled with various concentrations of SPIO nanoclusters for 12 h, these cells were fixed with 4 % paraformaldehyde for 0.5 h. Then, Perl’s stain A mixture was added (Leagene, China) into the wells for another 0.5 h, and then cells were washed with phosphate-buffered saline (PBS) for three times. Following, the cells were stained using Perl’s stain B (Leagene) for approximately 1 min. Finally, these staining cells were imaged and captured using a phase-contrast reverse microscopy (Nikon, Japan).

### Cellular MRI

The three types of cells were labeled with various concentrations of SPIO nanoclusters for 24 h. After washing with PBS, the cells were resuspended with 0.2 ml culture medium containing 2 % of agarose in Axygen PCR tubes to prepare MRI phantom, and the relaxation images were captured using a 9.4-T MR scanner (Bruker 94/20, Germany). The negative control was those of unlabeled cells. The intensity of the MRI images was measured with ImageJ (NIH, USA).

### MTS Assay

Cell viability was measured using MTS assay (Promega, USA). The three types of cells were seeded in 96-well plates at a density approximate 1 × 10^4^ cells/well and labeled with various concentrations of SPIO nanoclusters for 24 h and even to 36 and 48 h. MTS (20 μl/well) was added and incubated for 3~4 h at 37 °C. Finally, the absorbance density at 490 nm of formazan products was quantified with a spectrophotometer system (Mutiscan, Thermo, USA).

### LDH Assay

The three types of cells were seeded in 96-well plates at a density approximate 1 × 10^4^ cells/well respectively and incubated with various concentrations of SPIO nanoclusters for 24 h. LDH release was measured in cell-free medium following the manufacturer’s instructions (Beyotime, China).

### Annexin V/PI Staining FCS Assay

The three types of cells were seeded at 6 × 10^5^ cells/well in six-well plates. After 24-h labeling with or without SPIO nanoclusters, the cells were collected and resuspended, stained with Annexin V/PI assay kit (Beyotime), and measured with a flow cytometer (LSR-II, BD Biosciences, USA). The data were analyzed using FlowJo 6.7.1 software (Tree Star Inc., USA). Early apoptotic cells were stained with Annexin V, but without propidium iodide (PI). Late apoptotic cells were stained with both Annexin V and PI. The necrosis cells were only stained by PI. Each determination was based on the mean fluorescence intensity of at least 1 × 10^4^ events.

### Western Blotting Analysis

Cell protein were separated by sodium dodecyl sulfate polyacrylamide gel electrophoresis and transferred to polyvinylidene fluoride membranes, blocked with 5 % nonfat milk for 1 h, and then were incubated with specific anti-ferritin light chain (1:1000, Abcam, USA), anti-β-actin (1:2000, R&D, USA), and anti-cleaved caspase 3 (1:1000, CST, USA) antibodies overnight at 4 °C. Next, the membranes were incubated with the anti-rabbit secondary antibody (1:10,000, R&D). Finally, they were detected using chemiluminescence X-ray film. The expression of β-actin was used as control.

### Statistical Analysis

All data were displayed as mean ± standard deviation (SD) from a least three independent experiments. Statistical comparisons between different treatments were conducted using an unpaired Student’s *t* test with SPSS 18.0 software. *P* value is considered to be significant alteration when it is lower than 0.05.

## Results and Discussion

### Synthesis and Characterization of SPIO Nanoclusters

**Fig. 1:**
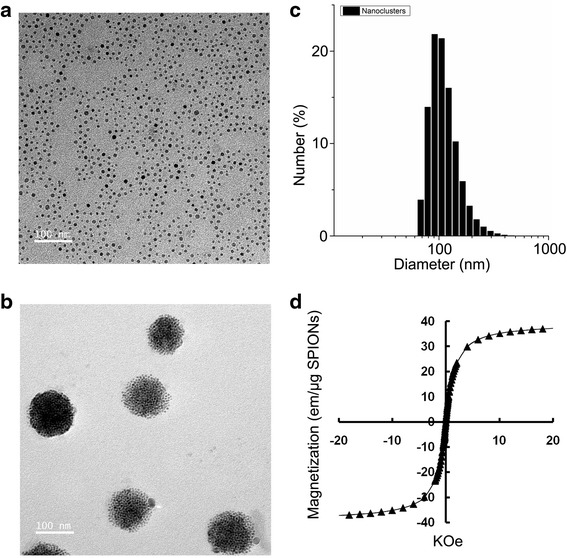
Synthesis and characterization of the SPIO nanoclusters. **a** The size of the monodisperse SPIO nanocrystals is detected using TEM. **b** The size of the SPIO nanoclusters is detected using TEM. **c** The size distribution of the SPIO nanoclusters is detected using dynamic light scattering. **d** Superparamagnetism of the SPIO nanoclusters

Our previous studies showed that the amphiphilic polycation PEI coated dozens of SPIO nanoclusters into a cluster which presented higher MRI sensitivity [25]. According to this, in the current study, the SPIO nanoclusters were prepared following a typical representative synthetic procedure with minor modification [6, 26, 27]. Firstly, the monodisperse SPIO nanocrystals were produced with a narrow size distribution which was 8.4 ± 2.3 nm using TEM (Fig. 1a). The SPIO nanocrystals were small enough to harbor superparamagnetism for MRI [25]. Then, hydrophobic SPIO nanocrystals produced SPIO nanoclusters with a controlled clustering structure under the help of Alkyl-PEI (115.3 ± 40.23 nm in size) (Fig. 1b, c). To evaluate the stability of the nanoclusters, surface charge and size distribution were examined using a Zetasizer Nano system. The zeta potential of the SPIO nanoclusters was 31.8 ± 2.6 mV, which was sufficient to maintain a stable formulation. As expected, the positively charged SPIO nanoclusters remained stable in PBS suspension with no signs of further aggregation for over 1 year, which was helpful in maintaining the superparamagnetic properties (Fig. 1d) and promoting the efficiency of cell labeling [6, 20].

### Cellular Uptake of SPIO Nanoclusters

**Fig. 2:**
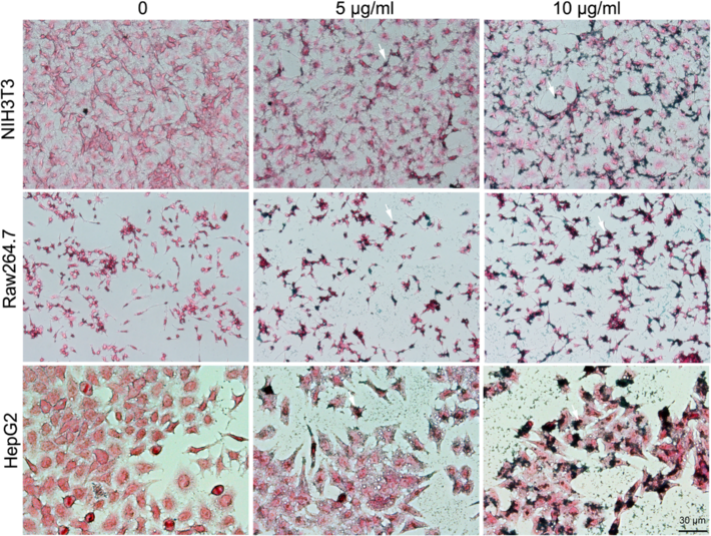
Cellular uptake of the SPIO nanoclusters. Cellular uptake of the SPIO nanoclusters (5 and 10 μg/ml) in NIH3T3, Raw264.7, and HepG2 cells is detected using Perl’s Prussian blue staining

The efficiency of cellular uptake is important for cell labeling and tracking. Cellular uptake of the SPIO nanoclusters was evaluated using Perl’s Prussian blue staining. A fibroblast cell line (mouse NIH3T3 cells), a macrophage cell line (mouse Raw264.7 cells), and an endothelial cell line (human hepatic HepG2 cells) were treated with 5 or 10 μg/ml SPIO nanoclusters for 12 h, and then stained with Prussian blue reagents. It was found that all the three types of cells could be labeled by the SPIO nanoclusters as detected using a phase-contrast reverse microscopy. The cellular uptake amount of the SPIO nanoclusters increased with the nanoclusters concentrations, and there was no significant difference among these cells (Fig. 2). Consistent with other reports, SPIO nanoclusters coated with a cationic polymer, such as PLL, display a better cell labeling efficiency than those nanoparticles with neutral or negative charge on their surface such as Feridex being modified with dextran [1, 28]. The low molecular weight Alkyl-PEI-SPIO nanoclusters are proved to have high efficiency on cellular uptake.

### MRI for Labeled Cells

**Fig. 3:**
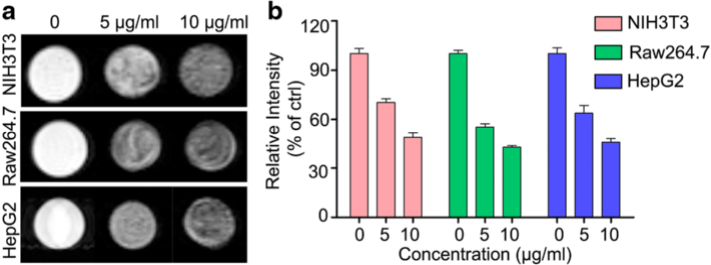
Cellular MRI performance of the SPIO nanoclusters as a contrast agent. **a** *T*_2_ relativity images for the SPIO nanoclusters labeling NIH3T3, Raw264.7, and HepG2 cells. **b** Relative intensity of MRI images for these three cell labeling

As one of the best noninvasive approach in medical imaging, MRI has several advantages including without exposure to X radiation, excellent spatial resolution, and good signal intensity contrast [8]. MRI also has been a useful tool in studying cell labeling with contrast agents [6]. As SPIO nanoclusters are *T*_2_-weighted MRI contrast agents, the darker *T*_2_-weighted images revealed the higher efficiency of SPIO nanoclusters labeling cells. To estimate the potential of the low molecular weight Alkyl-PEI-SPIO nanoclusters as MRI contrast agents, the *T*_2_ relaxivity MRI images of the nanoclusters were captured after labeling NIH3T3, Raw264.7, and HepG2 cells. The three types of cells incubated with the SPIO nanoclusters for 24 h were harvested and then imaged using a 9.4-T MRI scanner. With increasing concentrations of the SPIO nanoclusters, the contrast intensity of labeled cells was significantly decreased in *T*_2_-weighted MRI images in the three types of cells (Fig. 3). The cells labeled with the SPIO nanoclusters resulted in weaker signal intensity compared to those of the unlabeled cells, suggesting that the SPIO nanoclusters harbor excellent performance as an MRI contrast agent.

### The Biocompatibility of SPIO Nanoclusters

**Fig. 4:**
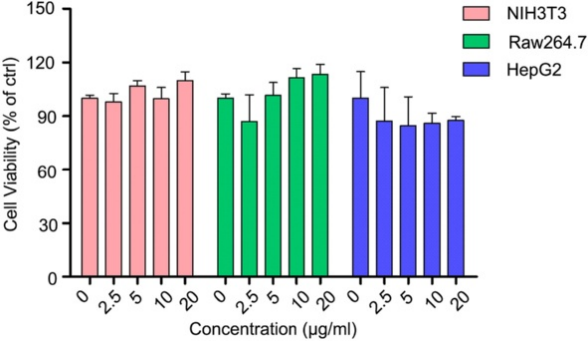
Effect of the SPIO nanoclusters on cell viability. The cell viability of NIH3T3, Raw264.7, and HepG2 cells labeled with the SPIO nanoclusters (2.5, 5, 10, and 20 μg/ml) at 24 h using MTS assay

SPIO nanoclusters have been applied in biomedical field due to their good biocompatibility in vitro and in vivo [29, 30]. For cell labeling, the biocompatibility of the internalized nanoparticles should be carefully evaluated. To investigate the potential cytotoxicity of the low molecular weight Alkyl-PEI-SPIO nanoclusters, cell viability was examined using MTS assay which measured mitochondrial NAD(P)H-dependent oxidoreductase activity. The three types of cells, NIH3T3, Raw264.7 and HepG2, were treated with various concentrations (2.5, 5, 10, and 20 μg/ml) of the SPIO nanoclusters for 24 h, and the MTS assay was performed. No significant cytotoxicity was observed in the three types of cells at any concentration examined (Fig. 4). We further performed this assay at 36 and 48 h, which also presented no significant decrease of cell viability (see in the Additional file 1: Figure S1). These data indicate that SPIO nanoclusters are with good biocompatibility at least in cell proliferation and mitochondrial function.

**Fig. 5:**
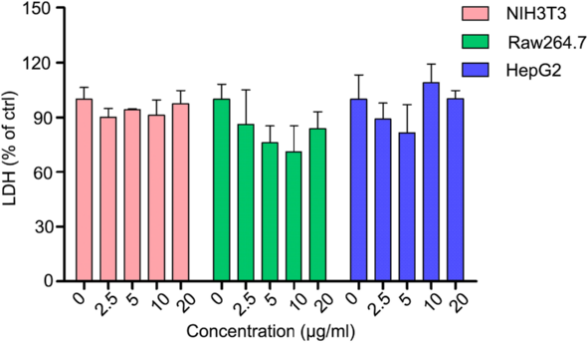
Effect of the SPIO nanoclusters on LDH release. The SPIO nanoclusters (2.5, 5, 10, and 20 μg/ml) label NIH3T3, Raw264.7, and HepG2 cells for 24 h, which do not disrupt cell membrane as detected using LDH assay

Apart from MTS assay for cell viability, LDH assay was performed to examine the biocompatibility of the nanoclusters. LDH is an enzyme existing in all cells, which will be rapidly released once the cell membrane damaged [31]. The toxicity on cell membrane could be determined by measuring the release of LDH into the cell culture supernatants. After labeled with or without SPIO nanoclusters for 24 h, LDH released from the cells was examined. As expected, similar to the results of MTS assay, the SPIO nanoclusters at all concentration examined did not increase the release of LDH in the culture medium compared to that of the unlabeled cells (Fig. 5). These results indicate that there is no damage of cell membrane when the SPIO nanoclusters label cells.

**Fig. 6:**
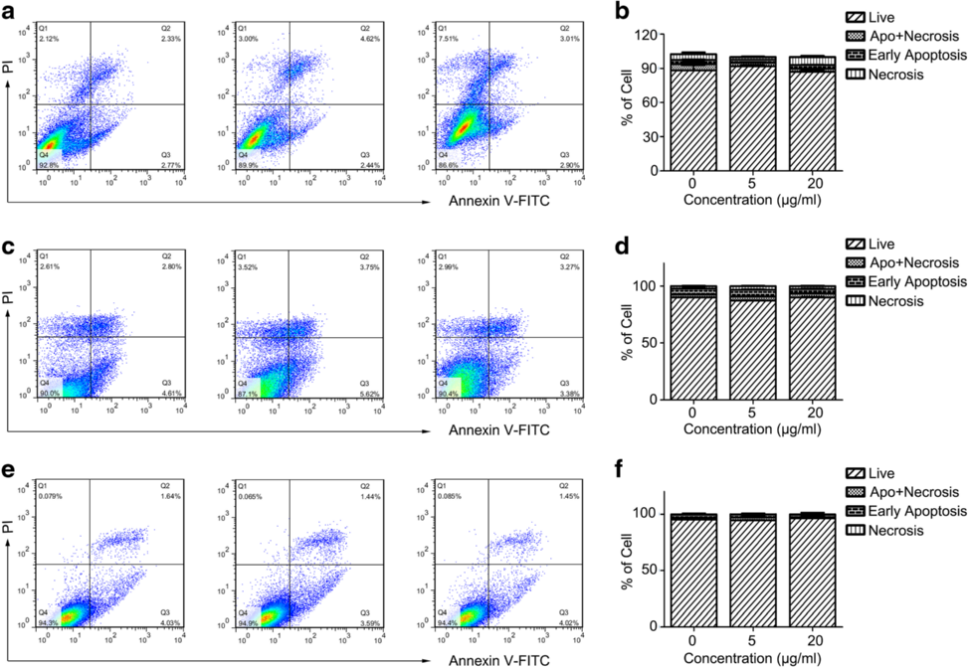
Effect of the SPIO nanoclusters on cell fate with Annexin V/PI staining FCS analysis. The SPIO nanoclusters (5 and 20 μg/ml) labeling for 24 h do not induce any significant apoptosis or necrosis compared to control in NIH3T3 (**a**, **b**), Raw264.7 (**c**, **d**), and HepG2 (**e**, **f**) cells using FCS assay

**Fig. 7:**
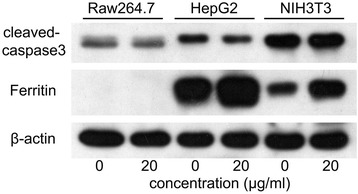
Effect of the SPIO nanoclusters on the expression of cleaved caspase 3 and ferritin proteins. Cleaved caspase 3 and ferritin expression after the SPIO nanoclusters (20 μg/ml) labeling for 24 h in Raw264.7, HepG2, and NIH3T3 cells is detected using western blotting assay

Furthermore, we studied the survival of these cells after labeling with the Alkyl-PEI-SPIO nanoclusters using Annexin V/PI FCS analysis. PI is permeable if cell membrane integrity is damaged, which can help us to tell alive cells, apoptosis, or necrosis [32]. At an early stage of apoptosis, apoptotic cells maintain membrane integrity, prohibiting PI from entering the cells. Annexin V translocates from the cell inner to the outer membrane, as it has high affinity to phosphatidylserine externalized particularly in the apoptotic cell membrane. Annexin V conjugation with the fluorescein isothiocyanate facilitates assay by FCS. As shown in Fig. 6, the SPIO nanoclusters did not induce any significant apoptosis or necrosis of NIH3T3 (Fig. 6a, b), Raw264.7 (Fig. 6c, d), or HepG2 (Fig. 6e, f) cells compared to the untreated cells. Moreover, we confirmed these results using western blotting assay. As one member in cysteine-aspartic acid protease (caspase) family, caspase 3 plays a key role in apoptosis. When caspase 3 is cleaved, the activation form triggers apoptosis process [33]. SPIO nanoclusters (20 μg/ml) did not increase the amount of cleaved caspase 3 in any cell lines tested (Fig. 7). All these data demonstrate that the SPIO nanoclusters present excellent biocompatibility in universal cell models.

Iron plays an important role in cells since it is essential in many processes such as oxygen storage and transport, photosynthesis, nitrogen fixation, and DNA synthesis. Importantly, cells have developed mechanisms to store the toxic iron ions which are not required for immediate metabolism and make them into a nontoxic form. As a protein sequestrating iron, the ferritin protein was measured in the present study. The level of ferritin protein was significantly induced by the SPIO nanoclusters in HepG2 and NIH3T3 cells, which was considered to be a response to keep iron hemostasis and cell survival [34, 35], while we did not observe ferritin expression in Raw264.7 cells, which might resulted from any other cell defense systems, such as glutathione [36] or autophagy [37], to protect the cells from iron overload (Fig. 7). Nevertheless, these results indicate that ferritin protein might play a protective role when SPIO nanoclusters label cells.

## Conclusions

In the present study, we developed SPIO nanoclusters consisting of iron oxide core wrapped within Alkyl-PEI. The Alkyl-PEI-SPIO nanoclusters are very stable without any aggregation for more than 12 months as the surface charge of SPIO nanoclusters is higher than 30 mV. Furthermore, the SPIO nanoclusters can be successful internalized in different types of cells. Significantly, SPIO nanoclusters show good performance as a contrast agent for cellular MRI. These data indicate that the SPIO nanoclusters have potential application in cell labeling and tracking. With careful attention on biosafety of the SPIO nanoclusters, the biocompatibility of the SPIO nanoclusters was studied using MTS, LDH, FCS, and western blotting assays, and there is no significant cytotoxicity in fibroblast cell lines, macrophage cell lines, or hepatic endothelial cell lines. We further observed that ferritin might protect cells from overload iron that leaks from SPIO nanoclusters. Therefore, our study provides a potential magnetic nanoclusters system with good biocompatibility for the universal cell labeling and MRI tracking.

## Additional file

Additional file 1: Effect of SPIO nanoclusters on cell viability. The cell viability of NIH3T3, Raw264.7, and HepG2 cells labeled with the SPIO nanoclusters (2.5, 5, 10, and 20 μg/ml) at 36 and 48 h using MTS assay.

## Abbreviations

caspase: cysteine-aspartic acid protease
FCS: flow cytometry
LDH: lactase dehydrogenase
MRI: magnetic resonance imaging
MTS: 3-(4,5-dimethylthiazol-2-yl)-5-(3-carboxymethoxyphenyl)-2-(4-sulfophenyl)-2H-tetrazolium
PBS: phosphate-buffered saline
PEI: polyethylenimine
PI: propidium iodide
PLL: poly-l-lysine
SPIO: superparamagnetic iron oxide
SQUID: superconducting quantum interference device
TEM: transmission electron microscope
