# Supplementary material for: Self-Assembled Superparamagnetic Iron Oxide Nanoclusters for Universal Cell Labeling and MRI
Source: Nanoscale Res Lett. 2016 May 23;11:263. doi: 10.1186/s11671-016-1479-5 (PMC4877342; doi:10.1186/s11671-016-1479-5)
Supplement: Additional file 1: — Effect of SPIO nanoclusters on cell viability. The cell viability of NIH3T3, Raw264.7, and HepG2 cells labeled with the SPIO nanoclusters (2.5, 5, 10, and 20 μg/ml) at 36 and 48 h using MTS assay. [file 11671_2016_1479_MOESM1_ESM.doc]

Additional files, Fig. S1


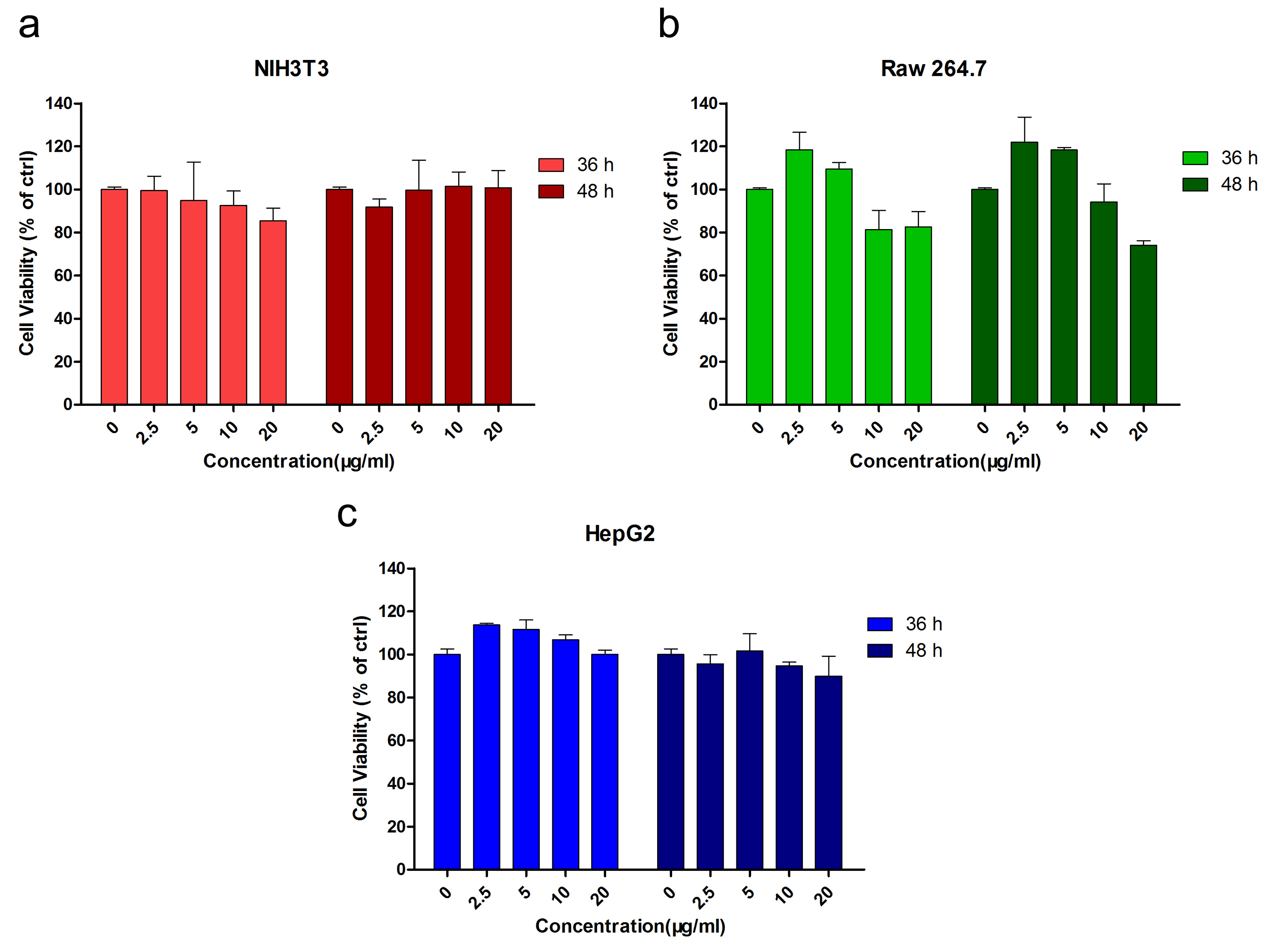


**Fig. S1** Effect of SPIO nanoclusters on cell viability. The cell viability of NIH3T3, Raw264.7, and HepG2 cells labeled with the SPIO nanoclusters (2.5, 5, 10, and 20 μg/ml) at 36 and 48 h using MTS assay.
